# Intrapericardial carboplatin in the management of malignant pericardial effusion in breast cancer: a pilot study

**DOI:** 10.1007/s00280-019-03897-0

**Published:** 2019-06-27

**Authors:** Mie Kotake, Hisao Imai, Kyoichi Kaira, Tomomi Fujisawa, Yasuhiro Yanagita, Koichi Minato

**Affiliations:** 1 Division of Respiratory Medicine, Gunma Prefectural Cancer Center, 617-1, Takahayashinishi, Ota, Gunma 373-8550 Japan; 2 Department of Respiratory Medicine, Comprehensive Cancer Center, International Medical Center, Saitama Medical University, Saitama, Japan; 3 Division of Breast Oncology, Gunma Prefectural Cancer Center, Ota, Japan

**Keywords:** Malignant pericardial effusion, Acute pericarditis, Breast cancer, Intrapericardial carboplatin, Catheter drainage

## Abstract

**Purpose:**

Malignant pericarditis is observed in 5.1–7.0% of all cases of acute pericarditis, and malignant pericardial effusion (MPE) can lead to cardiac tamponade in the later stages of cancer. Breast cancer is the second most common primary cancer associated with MPE, but the efficacy and safety of intrapericardial carboplatin (CBDCA) have never been evaluated in breast cancer. In this study, we assessed the clinical significance of intrapericardial CBDCA following catheter drainage in patients with breast cancer-related MPE.

**Methods:**

A catheter was inserted percutaneously into the pericardial space under echocardiographic guidance. After complete drainage, 150 mg of CBDCA was instilled into the pericardial space through the catheter.

**Results:**

Eight patients with symptomatic breast cancer-related MPE were treated at the Gunma Prefectural Cancer Center, between July 2010 and March 2016. One month after treatment, 100% of MPE was controlled. The median survival time from the recurrence of breast cancer until death or study follow-up was 2336 days (range 293–3937 days), while that from intrapericardial CBDCA administration until death or study follow-up was 552 days (range 35–1673 days). Grade 1 fever, nausea, hypotension, fatigue, and chest discomfort were observed in one patient (12.5%) after intrapericardial CBDCA administration.

**Conclusions:**

We found that intrapericardial administration of CBDCA after catheter drainage appears to be safe and effective in managing breast cancer-associated MPE. As the number of patients in this study was small, further studies are warranted to determine the safety and efficacy of intrapericardial CBDCA in the management of breast cancer-related MPE.

## Introduction

Malignant pericarditis is observed in 5.1–7.0% of all cases of acute pericarditis [1]. Malignant pericardial effusion (MPE) can develop into cardiac tamponade, which is a life-threatening disorder [2, 3]. Breast cancer is the second most common primary cancer associated with MPE [4]. Previous studies have demonstrated the efficacy of pericardiocentesis and extended catheter drainage is inadequate to prevent re-accumulation of MPE [5–7]. The intrapericardial instillation of various sclerosants such as tetracycline or doxycycline [8], tetracycline [9], cisplatin [10], thiotepa [11–13], mitomycin C [14], bleomycin [15], mitoxantrone [16, 17], minocycline [18], aclarubicin [19], and OK-432 [20] have been reported to be effective in controlling MPE. However, these studies examined a broad range of malignancies, and did not include a sufficient number of breast cancer patients with MPE to establish the most suitable sclerosants specific to this category. In several kinds of malignancies, including breast cancer, tetracycline, cisplatin, and thiotepa were effective, but caused serious complications [9–13]. The efficacy and safety of intrapericardial carboplatin (CBDCA) have never been evaluated in breast cancer. In this study, we assessed the clinical significance of intrapericardial CBDCA following catheter drainage in patients with breast cancer-related MPE.

## Patients and methods

### Patients

Patients with breast cancer-related symptomatic MPE were included in the study based on the following criteria: (i) symptoms caused by MPE histologically and/or cytologically defined as a result of breast cancer, (ii) 20 years of age or older, (iii) ECOG performance status (PS) 0–3, and (iv) white blood cells ≥ 2000/mm^3^, hemoglobin ≥ 8.0 g/dL, Platelets ≥ 50,000/mm^3^, aspartate aminotransferase (AST)/alanine amino transferase (ALT) < 5 times upper limit of normal (ULN), total bilirubin < 3 times ULN, and creatinine < 3 times ULN. Patients were excluded based on serious comorbidity, pregnancy or lactation, active infections, or coagulation disorders.

### Treatment method

A catheter was inserted percutaneously into the pericardial space under echocardiographic guidance. After the effusion was completely drained, 150 mg of CBDCA dissolved in 20 ml saline was instilled into the pericardial space through the drainage catheter. The catheter was then clamped and reopened after 2 h. When the drainage volume reached less than 50 ml/day, the catheter was removed. In cases where the catheter could not be removed within 7 days of treatment, CBDCA was administered a second time.

### Statistical analysis

Clinical characteristics, MPE control rate at 1 month after treatment, recurrence, complications, survival, time-to-drainage tube removal, and toxicity were assessed based on medical records. Toxicity was judged according to Common Terminology Criteria for Adverse Events (CTCAE) version 4.0. We used a Kaplan–Meier analysis of time-to-event data to estimate median event times. All analyses were performed using GraphPad Prism 8. The protocol complied with the Declaration of Helsinki. Our institution approved this study and all patients provided written informed consent.

## Results

### Patient characteristics

Eight patients with breast cancer-related symptomatic MPE were treated at the Gunma Prefectural Cancer Center between July 2010 and March 2016. Their characteristics and treatment outcomes are shown in Table 1. All were women with a median age of 59 years (range 47–73) who had previously received surgery. Six patients had invasive ductal carcinoma, one had invasive lobular carcinoma, and one had invasive mucinous carcinoma. Seven patients (87.5%) were diagnosed as having positive estrogen receptors (ER) and five (62.5%) were diagnosed as having human epidermal growth factor receptor 2 (HER2). Seven patients (87.5%) had a PS of 2–3, and one (12.5%) had a PS of 1 before the insertion of the catheter. CBDCA was administered once in seven patients and twice in one patient.

**Table 1:** Patient characteristics and treatment outcomes. All were at a stage of postoperative recurrence and all were discharged from the hospital after treatment

Case	Age	Histology	ER	HER-2	PS before the insertion of catheter	PS after the removal catheter	Duration of drainage (days)	No. of administration of carboplatin	Re-accumulation of MPE at 1 month after intrapericardial carboplatin administration	Systemic therapy after the control of MPE	Survival after recurrence (days)	Survival after intrapericardial carboplatin administration (days)
1	65	Invasive ductal carcinoma	(+)	(−)	3	1	8	1	(−)	(+)	3937	1437
2	51	Invasive mucinous carcinoma	(+)	(+)	3	1	6	1	(−)	(+)	3144	1359
3	73	Invasive lobular carcinoma	(+)	(+)	2	1	20	2	(−)	(+)	2767	837
4	47	Invasive ductal carcinoma	(+)	(−)	2	0	10	1	(−)	(+)	2330	1673
5	62	Invasive ductal carcinoma	(+)	(+)	1	1	6	1	(−)	(+)	1905	267
6	48	Invasive ductal carcinoma	(+)	(+)	3	1	6	1	(−)	(−)	1362	35
7	56	Invasive ductal carcinoma	(+)	(+)	2	1	5	1	(−)	(−)	676	92
8	68	Invasive ductal carcinoma	(−)	(−)	2	1	10	1	(−)	(+)	293	170

*ER* estrogen receptor, *MPE* malignant pericardial effusion, *HER-2* human epidermal growth factor receptor 2, *PS* performance status

### Efficacy

**Fig. 1:**
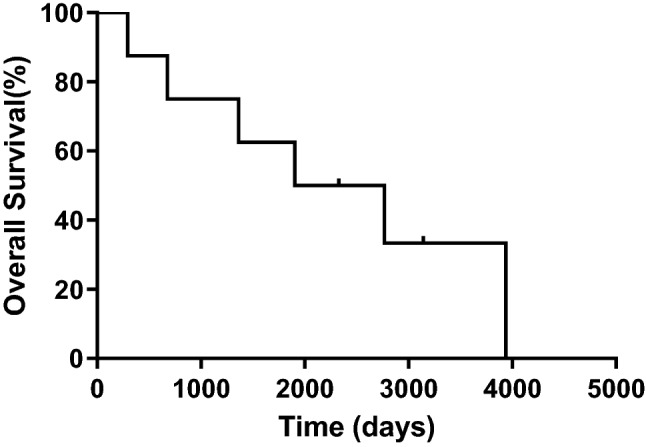
Survival time after recurrence of breast cancer until death or study follow-up. The median survival time from the recurrence of breast cancer until death or study follow-up was 2336 days (*n* = 8)

**Fig. 2:**
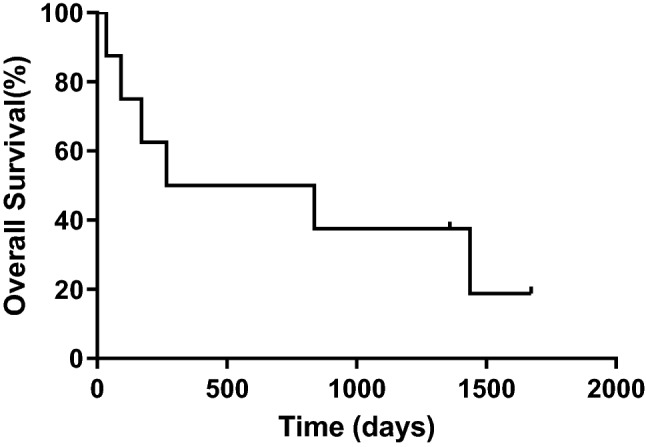
Survival time after intrapericardial carboplatin administration until death or study follow-up. The median survival time from the intrapericardial carboplatin administration until death or study follow-up was 552 days (*n* = 8)

The median duration of pericardial drainage was 7 days (range 5–20 days). The control rate of MPE at 1 month after this treatment was 100%. All patients had a PS of 0–1 after the removal of the catheter, and were discharged from hospital and survived 1 month after intrapericardial CBDCA administration. After the control of MPE, six patients received systemic therapy, with four receiving chemotherapy, four receiving hormonal therapy, and one receiving targeted therapy. The median duration from the instillation of CBDCA until the start of systemic therapy was 18 days (range 7–134 days) among four patients, while two continued systemic therapy during our study. The median survival time from the recurrence of breast cancer until death or study follow-up was 2336 days (range 293–3937 days) (Fig. 1). The median survival time from the intrapericardial CBDCA administration until death or study follow-up was 552 days (range 35–1673 days) (Fig. 2).

### Adverse events

Grade 1 fever, nausea, hypotension, fatigue, and chest discomfort were observed in one patient (12.5%) after intrapericardial administration of CBDCA. These were managed by supportive therapy. No patient experienced chest pain or arrhythmia, and we observed no significant complications or deaths resulting from treatment.

## Discussion

Malignant pericardial effusion is a potentially fatal complication of cancer. To our knowledge, this is the first study evaluating the efficacy and safety of intrapericardial administration of CBDCA to manage MPE in the patients with recurrent breast cancer. Moriya et al. [21] showed that the administration of 300 mg of CBDCA to the pericardial space in patients with non-small cell lung cancer (NSCLC) is effective for the control of MPE, with management achieved in nine out of ten patients. Their methods involved instilling 300 mg CBDCA and 100 mg lidocaine dissolved in 50 ml normal saline through the drainage catheter into the pericardial space, after which the catheter was clamped, then reopened after 40 min. In the present study, 150 mg CBDCA dissolved in 20 ml normal saline was infused via catheter, followed by clamping for 2 h. This dose was selected to suit the small body size of our patients (43.5–69.2 kg body weight). We also selected a longer clamping time due to the previous study’s finding that the high concentration of CBDCA in the pericardial effusion was enough to kill cancer cells 1.5 h after reopening the catheter, and that a low concentration of CBDCA in the plasma resulted in management of MPE with little systemic toxicity. This would help to ensure that intrapericardial CBDCA administration is suitable in patients for whom systemic chemotherapy is not appropriate.

In the present study, six patients received systemic therapy after the control of MPE with few adverse events, and the median survival time from the intrapericardial CBDCA administration until death or study follow-up was 552 days, which is longer than previous studies [4, 8–14, 17, 19] (Table 2). Patients with improved PS due to pericardial sclerosis received progressed systemic chemotherapy, contributing to the long survival time after intrapericardial CBDCA administration until death or study follow-up. Median duration of drainage was 9.5–10.5 days in NSCLC [21, 22]. Duration of drainage in the present study was comparable to that reported in previous studies.

**Table 2:** Pericardial sclerosis as management of malignant pericardial effusion in cancer patients, including those with breast cancer

Sclerosant	References	Successfully controlled/number of patients with malignancy treated (%)	Adverse events	Median survival (range)
Tetracycline or doxycycline	Maher et al. [8]	68/93 (73.1%)	Pain (*n* = 17), catheter plugged (*n* = 8), fever (*n* = 7), atrial fibrillation/flutter (*n* = 6), paroxysmal atrial tachycardia (*n* = 2), development of rub (*n* = 2), cardiac arrest before sclerosis (*n* = 2), infection (*n* = 1)	All: 98 days (1–1724), breast cancer: 131 days (6–1724)
Tetracycline	Shepherd et al. [9]	50/58 (86.2%)	Pain (*n* = 9), fever (*n* = 5), atrial fibrillation/flutter (*n* = 4), catheter plugged (*n* = 4), development of pericardial friction rub (*n* = 2), paroxysmal atrial tachycardia (*n* = 1)	133 days (3–1149)
Cisplatin	Maish et al. [10]	35/42 (83.3%)	Myocardial ischemia (*n* = 1)	2.8 ± 1.3 months
Thiotepa	Bishiniotis et al. [11]	19/19 (100%)	Atrial fibrillation (*n* = 2), vasovagal reaction (*n* = 1)	330 days (15–1040)
	Martinoni et al. [12]	30/33 (90.9%)	None	All: 115 days (22–1108), breast cancer: 272 days (47–1108)
	Colleoni et al. [13]	19/23 (82.6%)	Transient grade III thombocytopenia and leukopenia (*n* = 1), grade I leukopenia (*n* = 1)	4.5 months (1–26)
Mitomycin C	Lee et al. [14]	14/20 (70%)	Pericardial constriction (*n* = 1)	101 days
Bleomycin	Moya et al. [15]	14/18 (77.8%)	Mild fever (*n* = 5), atrial fibrillation (*n* = 3), retrosternal pain (*n* = 1), infection (*n* = 1)	Not reported
Mitoxantrone	Musch et al. [16]	15/16 (93.8%)	Loss of appetite (*n* = 1), leukocytopenia (*n* = 1)	Not reported
	Norum et al. [17]	2/5 (40%)	Not reported	122 days (28–294)
Minocycline	Lashevsky et al. [18]	10/14 (71.4%)	Severe chest pain (*n* = 7), pericardial injury (*n* = 2), vasovagal reaction (*n* = 1), transient fever (*n* = 1)	Not reported
Aclarubicin	Kawashima et al. [19]	5/5 (100%)	None	26 days (13–354)
OK-432	Imamura et al. [20]	3/3 (100%)	High fever, chills, chest pain (*n* = 3)	Not reported
Present study		8/8 (100%)	Fever (*n* = 1), nausea (*n* = 1), hypotension (*n* = 1), fatigue (*n* = 1), chest discomfort (*n* = 1)	552 days (35–1673)

Apodaca-Cruz et al. [5] reported a recurrence rate after pericardiocentesis as high as 33% in patients with MPE, including those with breast cancer. The recurrence rate of percutaneous prolonged catheter drainage in patients with MPE including breast cancer is 12–75% [6, 7]. However, the safety and efficacy of intrapericardial bleomycin compared with pericardial drainage have only been evaluated in lung cancer patients. In these patients, survival with MPE control (effusion failure-free survival, EFFS) at 2 months was not significantly different in patients with drainage alone versus intrapericardial bleomycin (29% versus 46%, one-sided *P *= 0.086 according to Fisher’s exact test) [23]. The recurrence rate of intrapericardial bleomycin for MPE, including breast cancer, was high (22.2%) and three patients (16.7%) developed severe complications [15]. In Table 2, we list trials evaluating pericardial sclerosis as management of MPE. Thiotepa, tetracycline, and cisplatin are capable of controlling 82.6–100% of MPE, but are associated with severe complications. Mitomycin C, bleomycin, and minocycline have been shown to have some efficacy. In this study, no patients had re-accumulation of MPE 1 month after pericardial sclerosis, and none experienced severe adverse events related to intrapericardial CBDCA administration. On the basis of this, we suggest that carboplatin is a suitable sclerosant for management of MPE.

There are several limitations to this study. As this was a pilot study, we did not specifically evaluate late cardiac complications, and our small patient population did not permit a randomized study to compare intrapericardial administration of CBDCA versus pericardial drainage alone. However, pericardial effusions complicate 1.9% of disseminated breast cancer [24]. Because of this low incidence, it would be difficult to achieve a prospective study evaluating pericardial sclerosis in an adequate number of patients with a single malignancy.

In conclusion, we found that intrapericardial instillation of CBDCA after catheter drainage appears to be safe and effective for the management of MPE associated with breast cancer. However, as the number of patients in this study was small, a large-scale phase II study is warranted to compare intrapericardial CBDCA administration with extended catheter drainage alone to manage MPE in breast cancer.
